# Remembrance of things past: The consequences of recurrent hypoglycaemia in diabetes

**DOI:** 10.1111/dme.14973

**Published:** 2022-10-21

**Authors:** Rory J. McCrimmon

**Affiliations:** ^1^ Systems Medicine, School of Medicine University of Dundee, Ninewells Hospital and Medical School Dundee UK

**Keywords:** glucose sensing, glycaemic variability, hypoglycaemia, impaired hypoglycaemia awareness, oxidative stress, review, type 1 diabetes

## Abstract

**Aims:**

People with type 1 and type 2 diabetes still frequently experience hypoglycaemia, which can be severe, leading to loss of consciousness. This review will examine the cellular consequences of recurrent hypoglycaemia.

**Methods:**

This review, based on the Dorothy Hodgkin Lecture given at the Diabetes UK 2022 annual symposium by the author, will discuss our current understanding of the mechanisms by which hypoglycaemia is detected and the consequences of recurrent exposure to hypoglycaemia.

**Results:**

Glucose‐responsive cells found in the periphery as well as multiple areas of the brain are organised in a classical sensori‐motor integrative network encompassing peripheral, hindbrain and hypothalamic components. The mechanism used by glucose‐responsive neurons to detect hypoglycaemia parallel those of the classical glucose sensor the pancreatic ß‐cell, namely in their use of glucokinase, K_ATP_ channels and AMP‐activated protein kinase. Recurrent exposure to hypoglycaemia results in a series of cellular adaptations that may be designed to increase the resilience of cells to future hypoglycaemia. This review also highlights how hypoglycaemia, as an oxidative stressor, may also exacerbate chronic hyperglycaemia‐induced increases in oxidative stress and inflammation, leading to damage to vulnerable brain regions.

**Conclusions:**

Impaired awareness of hypoglycaemia follows the adaptation of central glucose‐responsive neurons to repeated hypoglycaemia and may represent a form of memory called habituation. In diabetes, recurrent hypoglycaemia may have tissue consequences as a result of a profound disruption in the cellular response to a hypoglycaemic challenge that increases vulnerability to oxidative damage.

## 1 INTRODUCTION

In 1969 Dorothy Hodgkin solved the crystal structure for porcine insulin; work that led directly to the development of human and analogue insulins. In the 50 years since this discovery, we have seen a remarkable evolution in diabetes care, with further refinements of injectable analogues to produce more consistent basal insulin levels as well as faster acting insulin analogues that more closely mimic prandial insulin release. Insulin pump therapy has also advanced and is rapidly progressing, through combination with continuous glucose sensors and control algorithms, towards the introduction of the artificial pancreas into routine clinical practice.

Despite these advances, there remain a number of limitations to insulin therapy. Of these, the need to deliver insulin replacement peripherally which results in both systemic hyperinsulinaemia and a failure of insulin to dissipate when glucose levels fall (i.e. insulin release from the subcutaneous depot is not glucose dependent) is a major reason for the increased susceptibility of people with type 1 diabetes to hypoglycaemia. In people without diabetes, when glucose levels fall pancreatic ß‐cell insulin production is decreased and **α**‐cell glucagon secretion increased providing a stimulus to increase hepatic glucose production.^1^, ^2^, ^3^ If glucose levels fall further, the autonomic nervous system is activated with release of catecholamines both systemically and within peripheral tissues, as well as increased secretion of both anterior (e.g. cortisol) and posterior (e.g. vasopressin) pituitary hormones. In concert these changes broadly act to direct available glucose to essential organs, increase breakdown of other energy sources (glycogen, lipids, fat) and to alert the individual to hypoglycaemia through the generation of warning symptoms (autonomic and neuroglycopenic) that lead to behavioural change (feeding).^1^, ^2^, ^3^ In type 1 diabetes, this homeostatic response is profoundly disrupted. Almost all people with type 1 diabetes by around 5 years disease duration do not appear able to secrete glucagon in response to hypoglycaemia, while by 10 years disease duration the majority also have a suppressed autonomic response.^3^ Therefore, a combination of a failure of exogenously delivered insulin to dissipate and widespread defects in other aspects of the hormonal and symptomatic counterregulatory response means that hypoglycaemia is both far more frequent and can be severe in type 1 (and longer‐duration type 2) diabetes.^3^

Hypoglycaemia has a recognised morbidity and mortality.^4^ It is recognised as a significant limitation to individuals achieving recommended glycaemic targets.^1^ This review article is based on the Dorothy Hodgkin Lecture given at the 2022 Annual Diabetes UK conference. In it I will provide a brief overview of research conducted over the last 2–3 decades that has attempted to address three scientific questions, each driven by the need to understand why people with type1 diabetes are so susceptible to hypoglycaemia and why the symptom complex that classically alerts an individual to developing hypoglycaemia changes so much over time to the extent that many people were no longer aware of its onset. Because the lecture was based on work carried out in my own laboratory, the review article refers to much of the work we have contributed to this area, but as with so much of clinical science, there are of course many people and many research groups who have contributed to our current understanding of the mechanisms that underpin hypoglycaemic sensing in people with and without type 1 diabetes. The three main questions covered are: (i) where do we sense low glucose, (ii) how do we sense low glucose and (iii) what are the consequences of repeated exposure to hypoglycaemia?

## 2 WHERE DO SENSE LOW GLUCOSE?

It is now established that most vertebrate species have developed specialised cells within regions of the brain and periphery that are able to respond to changes in the glucose level to which they are exposed leading to the secretion of hormones (e.g. insulin) and/ or neurotransmitters (e.g. glutamate). The cells are linked either through neural pathways or via paracrine/endocrine hormones and acting together provide an integrated system for maintaining glucose homoeostasis.^5^

## 3 VENTROMEDIAL HYPOTHALAMUS

An example of one of these glucose‐sensing regions is the ventromedial hypothalamus (VMH). In a series of seminal studies it was shown that lesioning or directly microinjecting the non‐metabolisable glucose analogue, 2‐deoxy glucose, into the VMH in rodents stimulated a classical glucose counterregulatory response with rises in systemic levels of glucagon, adrenaline and noradrenaline.^6^, ^7^ Conversely, perfusing the VMH with glucose during systemic hypoglycaemia in rodents supressed the counterregulatory response.^8^ In work from our own laboratory, we were able to demonstrate that the medial nucleus of the amygdala (MAN) also contained glucose‐sensing neurons and contributed to the counterregulatory response to hypoglycaemia.^9^ The MAN was shown to contain glucose‐response neurons that expressed the pancreatic isoform of glucokinase, that lesioning the MAN suppressed, whereas 2‐DG infusion amplified the counterregulatory response to acute hypoglycaemia. Moreover, we were able to demonstrate that there were neural pathways directly linking the MAN and VMH that were active during hypoglycaemia.^9^

## 4 BRAIN GLUCOSE‐SENSING REGIONS

It is now recognised that glucose‐sensing neurons are found within a number of areas of the brain as well as in the periphery. The most well studied of these brain regions is the hypothalamus where glucose‐responsive neurons have been identified in the VMH, arcuate, lateral hypothalamus, paraventricular hypothalamus and supraoptic nucleus. Glucose‐sensing neurons have also documented in brain regions involved in motivation and reward such as the nucleus accumbens, amygdala, paraventricular thalamus, hippocampus and pre‐frontal cortex, and in the hindbrain (e.g. nucleus tractus solitarius) which coordinates functions that are fundamental to survival. As glucose is the primary fuel for all cells, the cardinal feature of glucose‐responsive neurons (as we will discuss below) is their ability to detect changes in glucose and then use that information to produce the appropriate counterregulatory response. As this response involves physiological, symptomatic and behavioural elements it has to be co‐ordinated. Watts and Donovan,^5^ have argued that discrete populations of glucose‐responsive neurons are organised in a classical sensori‐motor integrative network encompassing peripheral, hindbrain and hypothalamic components. In this hierarchical system, the forebrain is able to modulate the ‘reflex loops’ that exist in the hindbrain. This model is yet to be proven, and there remains much about the nature and detailed arrangements of its components parts that are still unknown. It did, however, provide the theoretical basis for interventions designed to recover defective glucose sensing such as ‘Dishabituation’ as I will describe later.

## 5 HOW DO WE SENSE LOW GLUCOSE?

Using the ventromedial hypothalamus (VMH) as an exemplar for a glucose‐sensing region, Sherwin proposed that glucose‐sensing neurons operated in a way that appeared to parallel the pancreatic islet.^10^ In this model, like pancreatic ß‐ and **α**‐cells, glucose‐excited and glucose‐inhibited neurons respond to higher and lower blood glucose levels respectively. As in the pancreatic ß‐cell, key components of the mechanism through which a change in glucose levels lead to an alteration in neural firing rates should therefore involve glucokinase, sulfonylurea receptor 1 (SUR1) ATP‐sensitive potassium (K_ATP_) channels and AMP‐activated protein kinase (AMPK).^10^ Consistent with this hypothesis, mice and humans with reduced glucokinase activity show an exaggerated response to hypoglycaemia,^11^ while glucokinase activation in hypothalamic glucose‐excited neurons reverses the hyperpolarising effect of low glucose.^12^ Subsequently, the increased ATP:AMP ratio with increased glucokinase activity results in closure of K_ATP_ channels, depolarising the neurons and increasing their firing rate.^13^

## 6 K_ATP_ CHANNEL

It was established a number of years ago that glucose‐sensing neurons ex vivo contained functional K_ATP_ channels^14^ and we were later able to show in a series of in vivo rodent studies that perfusion of the VMH with the K_ATP_ channel blocker glibenclamide suppressed,^15^ whereas perfusion with a SUR‐1‐selective K_ATP_ opener NN414 amplified^16^ the counterregulatory response to hypoglycaemia. Subsequently, we demonstrated that systemic delivery of the K_ATP_ channel opener NN414 amplified the counterregulatory response to hypoglycaemia in rodents with or without type 1 diabetes,^17^ paving the way for a study in humans with type 1 diabetes who had impaired awareness of hypoglycaemia.^18^ In this study, oral delivery of the non‐selective K_ATP_ channel opener, diazoxide significantly amplified the hormonal counterregulatory response to hypoglycaemia.^18^ Taken together with an extensive literature in in vitro and ex vivo systems, it seems clear that SUR1 K_ATP_ channels in glucose‐responsive neurons, as they do in the pancreatic ß‐cell, are integral to the transduction of the glucose signal.

## 7 AMP‐ACTIVATED PROTEIN KINASE

**FIGURE 1:**
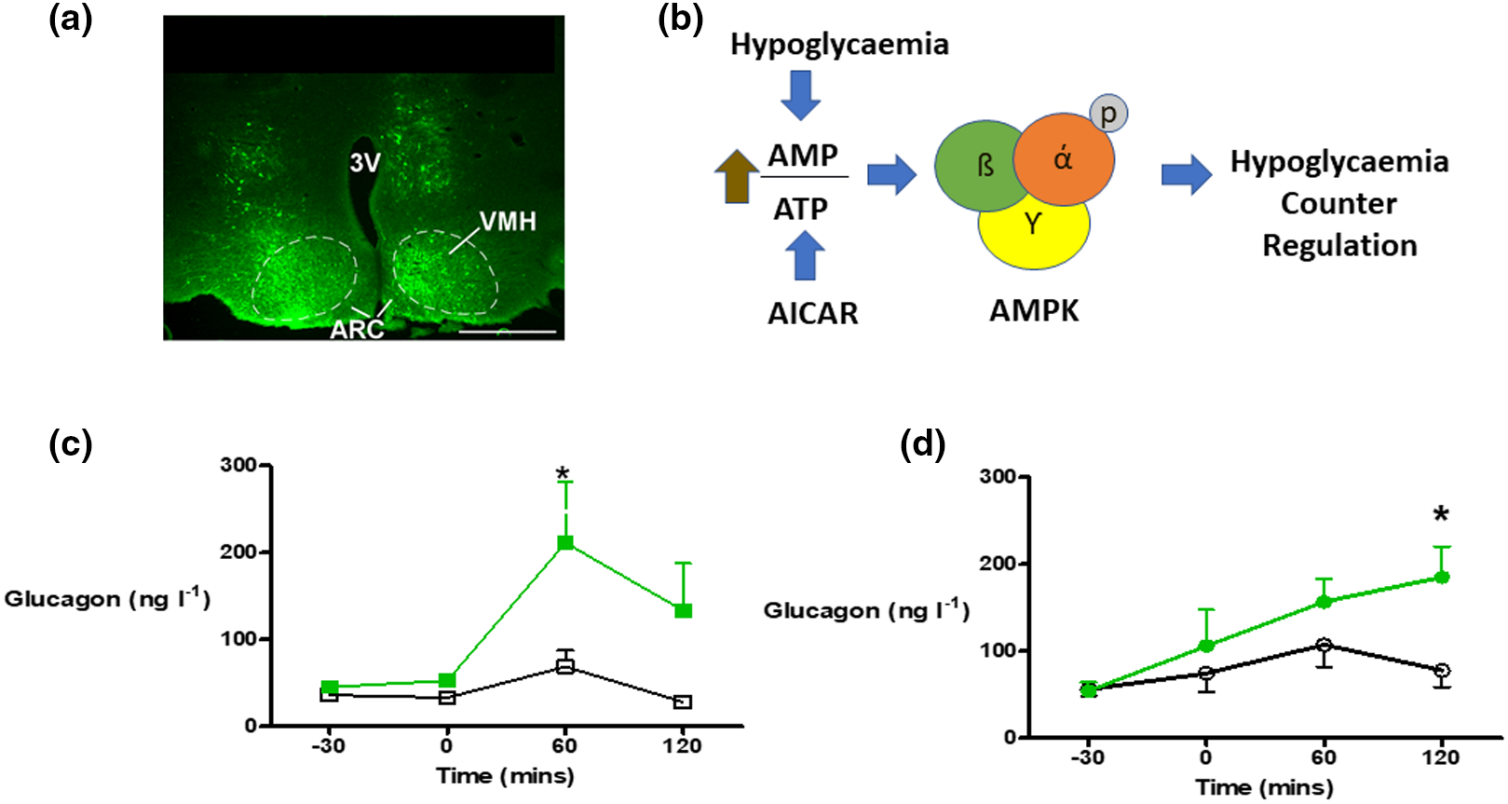
Role of AMPK in the detection of hypoglycaemia. (a) Brain section with immunohistochemical staining for green fluorescent protein (GFP) after microinjection of an adeno‐associated virus (AAV) containing GFP into the ventromedial hypothalamus (VMH). The arcuate (ARC) nucleus is also labelled. (b). Hypoglycaemia or 5‐aminoimidazole‐4‐carboxamide ribonucleotide (AICAR) increases the AMP:ATP ratio within the glucose‐sensing neuron resulting in activation and phosphorylation of AMPK, which leads to amplification of the counterregulatory response to hypoglycaemia. Pharmacological activation of AMPK by microinjection of AICAR into the VMH restores defective hypoglycaemia‐induced glucagon secretion in (c). Recurrently hypoglycaemic and (d). Chronically hyperglycaemic diabetic BB rats. In each of these studies, hypoglycaemia was induced in awake, unrestrained rats using the hyperinsulinaemic hypoglycaemic clamp technique with insulin infusion starting at time = 0 mins and the hypoglycaemic plateau (2.8 mmoL/L) maintained from 60 to 120 mins. VMH AICAR‐injected animals are represented by the green symbols and lines and the saline‐injected control group in open symbols/ black lines. *p < 0.05.

Another component of glucose sensing in the pancreatic ß‐cell is the energy sensor, AMP‐activated protein kinase (AMPK). In a series of studies in rodent models, we were able to demonstrate that pharmacological activation using 5‐aminoimidazole‐4‐carboxamide ribonucleotide (AICAR)^19^, ^20^ or adenoviral suppression of AMPK in the VMH^21^ amplified or suppressed the counterregulatory response to hypoglycaemia. Interestingly, VMH microinjection of AICAR restored the glucagon counterregulatory response to hypoglycaemia in the diabetic BB rat, a rodent model of autoimmune type 1 diabetes that, as with its human counterpart, develops a hypoglycaemic‐specific defect in glucagon secretion shortly after it develops type 1 diabetes (Figure 1).^22^

**FIGURE 2:**
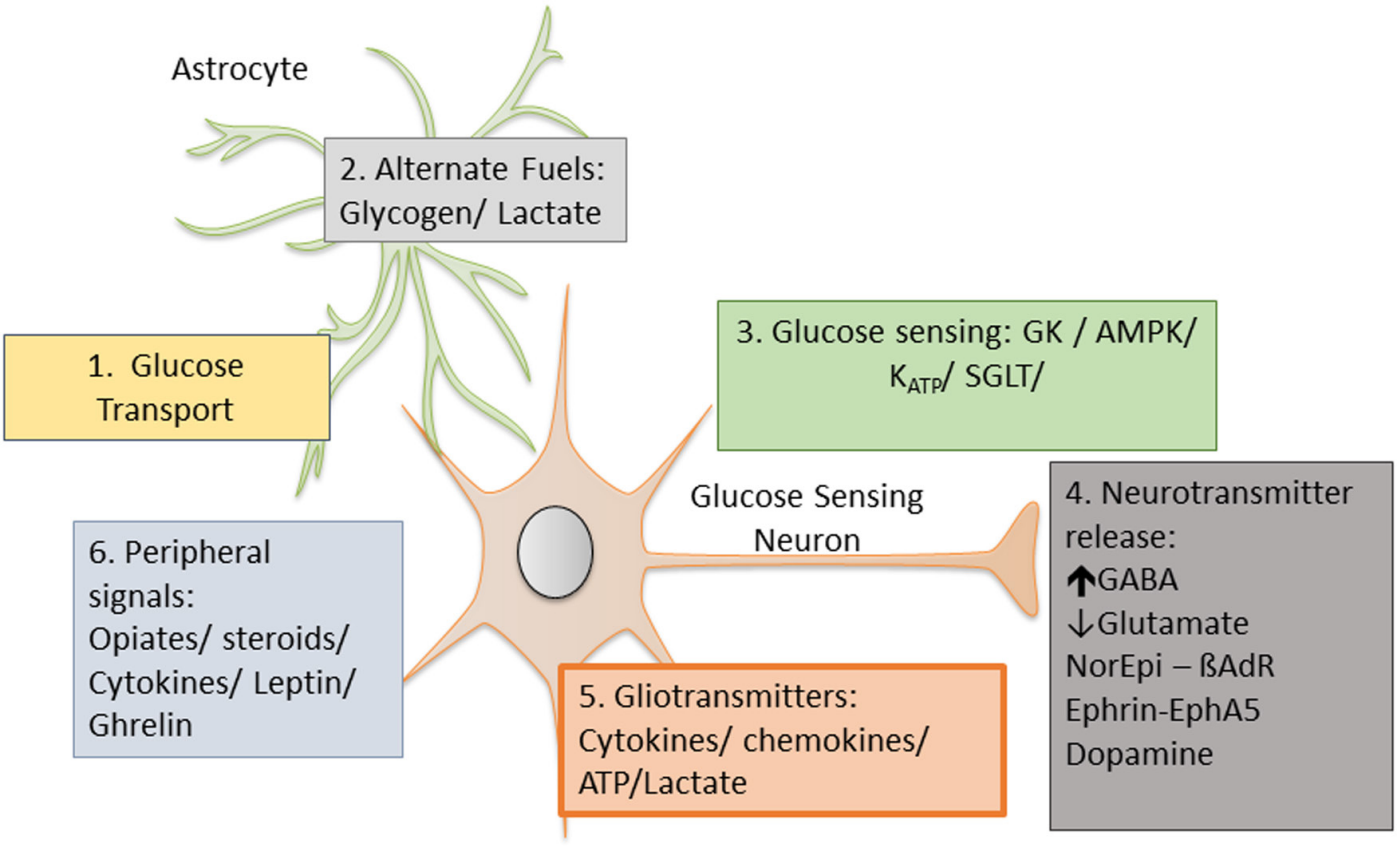
Potential mechanisms of cerebral adaptation to recurrent hypoglycaemia. These include: (1) An increase in glucose uptake; (2) metabolic switching to alternate fuel use; (3) an increase in the metabolism of glucose; (4) changes in or modulation of neurotransmitter release or action; (5) actions of locally released gliotransmitters; (6) external modulators. Adapted from.^40^

Once activated glucose‐excited neurons are believed to release the inhibitory neurotransmitter, gamma aminobutyric acid (GABA),^23^ while glucose‐inhibited neurons release the excitatory neurotransmitter, glutamate^24^; an interesting parallel with pancreatic ß‐ and **α**‐cells who co‐secrete these neurotransmitters with insulin and glucagon respectively.^25^, ^26^ A number of other transporters (e.g. GLUT‐2), membrane channels or enzymes that contribute to glucose sensing, such as sodium–glucose cotransporters (SGLTs), sweet taste receptors, Na^+^/K^+^ ATPase, K^+^ channels and nitric oxide synthase (NOS), Ephrins and beta‐adrenergic receptors have been described but are beyond the scope of this review (Figure 2). Readers are referred to a detailed review of this area by Stanley et al.^13^ Complex mechanisms also exist by which glucose‐sensing neurons in one brain region may modulate those in another. For instance, following our demonstration of a neural pathway involving urocortin III neurons from MAN to VMH,^9^ we were able to show that urocortin acting via corticotrophin‐releasing hormone receptor (CRHR) 2 in the VMH suppressed,^27^ whereas CRHR 1 receptor activation amplified the counterregulatory response to hypoglycaemia.^28^ Overall, our current understanding of the mechanisms through which the human body detects a falling glucose is that these signalling pathways are, at least in part, very similar to those employed by the pancreatic ß‐cell.

## 8 WHAT ARE THE CONSEQUENCES OF REPEATED EXPOSURE TO HYPOGLYCAEMIA?

The most well‐recognised consequence of recurrent hypoglycaemia for people with type 1 and insulin‐treated type 2 diabetes is the development of impaired awareness of hypoglycaemia which markedly increases the risk of severe hypoglycaemia. The seminal work of Heller established that exposure to hypoglycaemia resulted in a marked suppression of the symptomatic and hormonal counterregulatory responses to a subsequent episode of hypoglycaemia induced the following day.^29^ This finding has now been replicated in many human and rodent studies.^3^ The more frequent the exposure to hypoglycaemia the bigger the degree of suppression seen, while hypoglycaemia avoidance restores the counterregulatory response.^3^

## 9 RECURRENT HYPOGLYCAEMIA AND BRAIN GLUCOSE UPTAKE

This whole systems response to recurrent hypoglycaemia is mirrored in ex vivo electrophysiological studies where there is a left‐shift in glucose‐excited neurons so that they do not hyperpolarise until glucose levels fall further.^30^ This suggests that glucose‐responsive neurons are adapting to recurrent hypoglycaemia. The mechanisms underpinning this adaptation are not clear and multiple potential mechanisms have been proposed (Figure 2). A logical explanation might be that recurrent hypoglycaemia leads to an increase in glucose uptake or metabolism. Consistent with this are reports of increased hexokinase activity following recurrent hypoglycaemia,^31^ implying that glucose‐excited neurons were better able to maintain intercellular ATP:AMP ratios during subsequent hypoglycaemia and, hence, maintain GABAergic tone in the VMH.^32^ Moreover, recurrent hypoglycaemia in rodents increases expression of the glucose transporter, GLUT1, at the blood–brain barrier (BBB),^33^ while in vivo microdialysis of brain extracellular fluid reports higher glucose levels in rodents exposed to recurrent hypoglycaemia.^34^ In contrast, human studies using magnetic resonance spectroscopy or positron emission tomography (PET) have produced conflicting data about the effects of recurrent hypoglycaemia on cerebral glucose uptake and metabolism.^13^, ^35^ The disparity in this findings may reflect species differences, regional variation in glucose metabolism, or even differences in how neurons and glial cells individually respond to recurrent hypoglycaemia.^13^, ^35^, ^36^

## 10 RECURRENT HYPOGLYCAEMIA AND BRAIN ALTERNATE FUEL USE

Alternately, recurrent hypoglycaemia may induce cellular adaptations that allow lactate or ketones to be used as alternate fuels.^13^, ^35^ Lactate or ketone infusions suppress counterregulation to systemic hypoglycaemia in humans,^13^ and lactate transport and lactate metabolism by the brain are thought to be increased in both humans and rodents who have been exposed to recurrent hypoglycaemia^13^, ^35^ . Initial research also suggested that recurrent hypoglycaemia led to increased astrocytic glycogen storage (supercompensation), which could provide additional lactate during subsequent hypoglycaemia, although these findings were not replicated in later studies.^13^ Lactate might act to modulate the counterregulatory response to hypoglycaemia via suppression of AMPK^37^ and increased GABA release.^38^ However, other studies suggest that lactate may be insufficient to support metabolism during hypoglycaemia.^39^ Other putative mechanisms that may contribute to the development of impaired hypoglycaemia awareness include the actions of external neuronal modulators, such as opioids, serotonin, steroids, cytokines or urocortin, all of which have been shown to modulate the counterregulatory response to insulin‐induced hypoglycaemia and can affect changes in neurotransmitter synthesis or release, or changes in synaptic structure or alterations in neurotransmitter release and/or action.^3^, ^13^, ^40^

## 11 HABITUATION

More recently, our group have proposed that the whole‐organism response to recurrent hypoglycaemia represents a form of adaptive memory, referred to as ‘habituation’. Habituation is defined as a “reduction of the psychological, behavioural or physiological responses to a stimulus as a result of repeated or prolonged exposure”.^41^ Interestingly the model system often used to demonstrate habituation is the gill and siphon withdrawal reflex (GSWR) in Californica Aplysia (Sea Slug). Repeatedly inducing a GSWR leads eventually to the reflex response becoming suppressed, whereas it will recover over time if the stimulation is withheld. Based on this literature and the proposal by Watts and Donovan that glucose sensing by the brain also operated as a sensori‐motor response,^5^ we sought to determine whether recurrent hypoglycaemia in rodents and humans would demonstrate other cardinal features of a habituated response.

Our first approach was to examine whether we could restore counterregulatory responses to hypoglycaemia in animals that had been exposed to recurrent hypoglycaemia by introducing a ‘dishabituatory’ stimulus. Dishabituation is the introduction of a novel, usually strong stimulus, to a habituated response which will result in at least temporary restoration of the habituated response.^41^ In our first study, rodents who had been exposed to 4 weeks of recurrent hypoglycaemia were randomised to undergo either a single episode of high intensity training (HIT) on a treadmill or control. The following day hypoglycaemia was induced in all animals using a hyperinsulinaemic glucose clamp and counterregulatory hormone response compared. The rodents who had been exposed to the short HIT intervention were found to have a significantly greater counterregulatory response during subsequent hypoglycaemia, consistent with dishabituation.^42^ In a further study in rodents we found that cold exposure (4°C for 4 h) also had a similar dishabituatory effect.^43^ Subsequently, we have also been able to show in a proof of concept study that a single episode of HIT also significantly augments counterregulatory hormonal and symptomatic responses to hypoglycaemia in people with type 1 diabetes who have impaired awareness of hypoglycaemia.^44^ The mechanism by which dishabituation restores the counterregulatory response is unknown but may involve recovery of hypothalamic glutaminergic transmission.^42^

**FIGURE 3:**
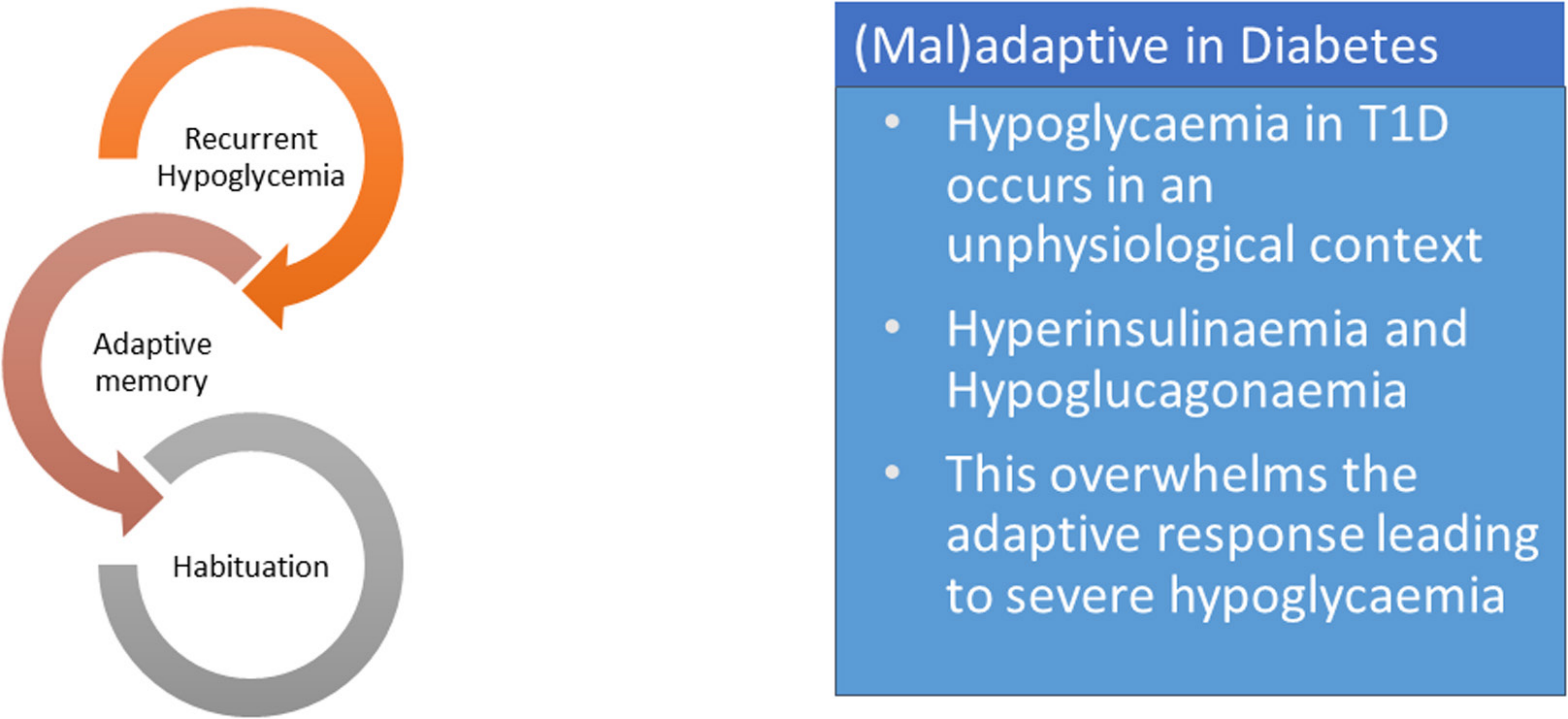
Habituation to recurrent hypoglycaemia. Recurrent hypoglycaemia initiates an adaptive memory response leading to habituation of the counterregulatory response. While this is adaptive at a cellular level it may be considered ‘maladaptive’ in type 1 diabetes, largely because the inability to switch off exogenous insulin that is being released continuously from a subcutaneous depot, and because of the hypoglycaemia‐specific defect in alpha cell‐derived glucagon release, means that hypoglycaemia is occurring in ‘unphysiological’ context and so can become severe leading to confusion or loss of consciousness.

In summary, it is apparent that recurrent hypoglycaemia leads to a series of adaptations that may be direct or indirect in specialised glucose‐sensing cells throughout the body, the net effect of which is to reduce the responsiveness of these cells to subsequent hypoglycaemia (a ‘left‐shift’ in the counterregulatory response to subsequent hypoglycaemia). The culmination of these changes in many different brain regions is the development of impaired awareness of hypoglycaemia in people with diabetes. This is considered an adaptive response that may develop through habituation and is likely to represent a mechanism to ensure the cells remain resilient to future periods of low glucose. This may also be considered ‘maladaptive’ in type 1 diabetes, largely because the inability to switch off exogenous insulin that is being released continuously from a subcutaneous depot, and because of the hypoglycaemia‐specific defect in alpha cell‐derived glucagon release, means that hypoglycaemia is occurring in ‘unphysiological’ context and so can become quite profound (Figure 3). As a consequence, recurrent hypoglycaemia in diabetes leads both to impaired awareness of hypoglycaemia and a greatly increased risk of severe hypoglycaemia.

## 12 GLYCAEMIC VARIABILITY AND COGNITIVE FUNCTION

More recently it has also been recognised that recurrent hypoglycaemia in diabetes may have long‐term consequences particularly on brain function. Epidemiological research on the long‐term impact of hypoglycaemia on the brain is limited, largely because most studies are only able to examine the association between severe hypoglycaemia and cognitive performance or brain structure. Meta‐analyses report that a history of severe hypoglycaemia is associated with a small negative effect on a number of different cognitive domains, especially with early‐onset type 1 diabetes.^45^ However, the 32‐year follow‐up of participants enrolled in the Diabetes Control and Complications Trial (DCCT)/Epidemiology of Diabetes Interventions and Complications (EDIC) reported that severe hypoglycaemia as well as higher HbA1c levels were independently associated with a greater rate of cognitive decline; collectively equivalent to 9.4 years accelerated brain aging.^46^

The brain is vulnerable to hypoglycaemia due to its high metabolic demand, reliance on glucose as a fuel, minimal fuel stores and reduced capacity to access alternate fuels because of systemic hyperinsulinaemia.^47^ Hypoglycaemia of sufficient severity to induce an isoelectric EEG will result in neuronal death in distinct areas of the brain such as the hippocampus; a brain region considered critical to human cognition and has an extensively researched role in memory function as well as processing speed and intelligence.^48^ We recently reported in a rodent model of chemically induced (Streptozotocin) type 1 diabetes that recurrent hypoglycaemia exacerbated the negative impact of chronic hyperglycaemia on a number of cognitive tasks that assessed hippocampal function.^49^ This was associated with evidence of lipid peroxidation and protein carbonylation in the hippocampus, markers of oxidative damage to the tissue.^49^

**FIGURE 4:**
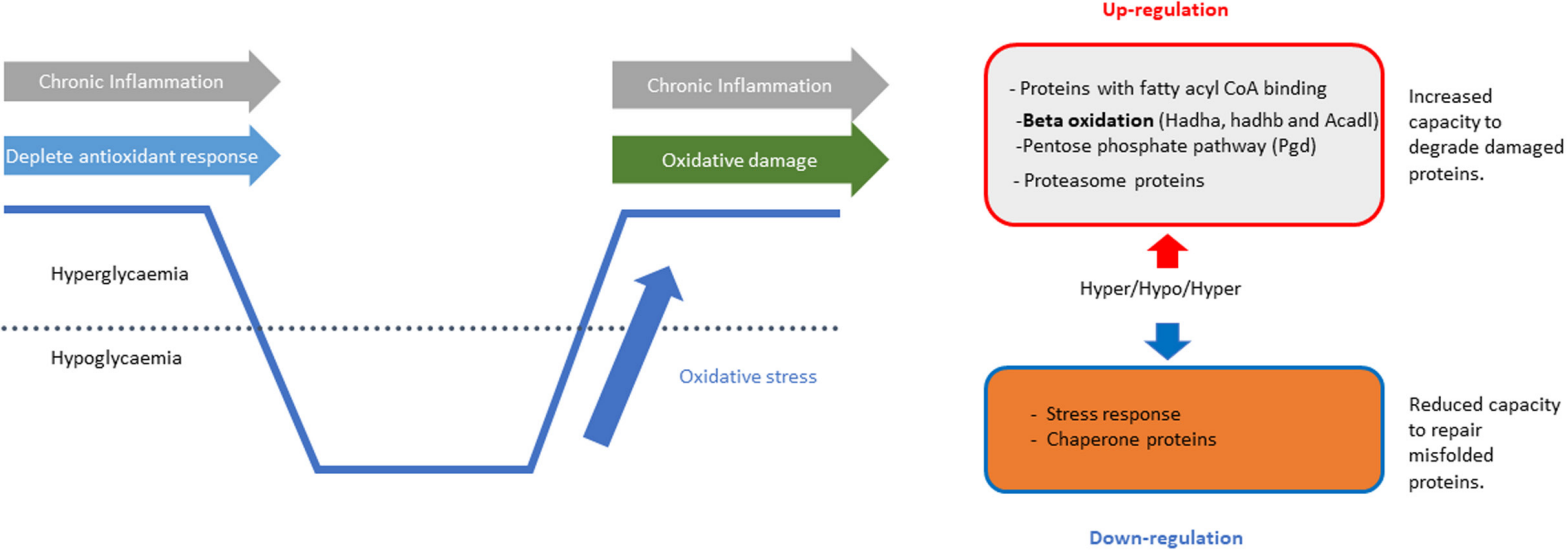
Impact of chronic hyperglycaemia, hypoglycaemia and hypoglycaemia recovery on the brain. Chronic hyperglycaemia, hypoglycaemia and post‐hypoglycaemic hyperglycaemia, result in excessive production of reactive oxygen species (ROS) leading to irreversible oxidative cell damage within the brain and contributing to accelerated cognitive decline. Proteomic studies of the hypothalamus show an up‐regulation of pathways identified significant up‐regulation of proteins involved in long‐chain fatty acid metabolism (predominantly beta oxidation), and components of the proteasome, suggesting an enhanced capacity for long‐chain fatty acid oxidation and the degradation of damaged proteins. Conversely, significant down‐regulation of proteins involved in mediating the stress response, including several heat shock proteins, was observed. Adapted from.^40^

Chronic hyperglycaemia,^50^ severe hypoglycaemia^49^ and glucose recovery from hypoglycaemia all stimulate reactive oxygen species (ROS) production, which in the latter case correlates directly with the degree of glucose rise during recovery from hypoglycaemia.^51^ Chronic hyperglycaemia has also been reported to impair antioxidant defence mechanisms.^52^, ^53^ Consequently, we proposed that increased glycaemic variability, encompassing chronic hyperglycaemia, hypoglycaemia and post‐hypoglycaemic hyperglycaemia, may result in excessive production of reactive oxygen species (ROS) leading to irreversible oxidative cell damage within the brain and contributing to accelerated cognitive decline.^49^ Recent unpublished data from our laboratory from animal models with and without type 1 diabetes studied using the hyperinsulinaemic glucose clamp combined with hypothalamic SILAC proteomics has revealed a profound disruption in the hippocampal cellular response to a hypoglycaemic challenge in type 1 diabetes that increases the vulnerability of the hippocampus to oxidative damage. Post‐hypoglycaemic hyperglycaemia in type 1 diabetes was associated with a down‐regulation of proteins mediating the stress response and reductive biosynthesis. This is likely to result in proteotoxic stress through a reduced ability of cells to maintain the correct folding of proteins damaged by the stress challenge, which may in turn lead to irreversible damage/modification to proteins or synapses between cells within crucial brain regions such as the hippocampus. These observations may in part explain the association between severe hypoglycaemia and accelerated cognitive deficits associated with suboptimally controlled type 1 diabetes (Figure 4).

## 13 CONCLUSION

In this review I have provided an outline of research performed in my own laboratory as well as a number of others that has sought to understand the mechanisms that underpin the physiological response to hypoglycaemia. Some of this has been discovery research, seeking to understand the basic biology of glucose sensing by the brain. To that extent, it is now broadly accepted that discrete populations of glucose‐responsive neurons are present within peripheral, hindbrain, hypothalamic and other brain regions that are organised in a classical sensori‐motor integrative network. Within these regions, we find glucose‐excited and glucose‐inhibited neurons that respond to high and low blood glucose levels, respectively and use signalling mechanism that are broadly similar to those found in the pancreatic ß‐cell with key components being glucokinase, sulfonylurea receptor 1 (SUR1) ATP‐sensitive potassium (K_ATP_) channels and AMP‐activated protein kinase (AMPK). The research programmes are also translational, seeking to understand why recurrent hypoglycaemia leads to suppression of the normal physiological counterregulatory response. Here current evidence supports the idea that this is as a result of a series of cellular adaptations, presumably designed to better preserve cell integrity during future hypoglycaemia. This understanding offers the opportunity of novel approaches to preventing or restoring defective counterregulation through interventions such as HIT. Finally, a literature is emerging that recurrent hypoglycaemia, when associated with increased glycaemic variability, may have longer term consequences in some tissues such as the brain. Oxidative damage as a result if an impaired cellular stress response to hypoglycaemia, particularly when there is a marked glucose excursion on recovery, may accelerate tissue aging. This highlights the importance not just of hypoglycaemia avoidance, but of achieving recommended glycaemic targets and careful management of glucose recovery from a hypoglycaemic event.

## FUNDING INFORMATION

The work of the McCrimmon Laboratory has been supported by grants from the JDRF, Diabetes UK, Tenovus Scotland, the Medical Research Council, the Wellcome Trust, the EU Innovative Medicines Initiative and the Helmsley Trust.
